# Apremilast in der Therapie der palmoplantaren Pustulose: Eine Fallserie

**DOI:** 10.1007/s00105-020-04676-2

**Published:** 2020-09-02

**Authors:** Nina Frischhut, Gudrun Ratzinger

**Affiliations:** Univ.-Klinik für Dermatologie, Venerologie und Allergologie, Medizinische Universität Innsbruck, Anichstr. 35, 6020 Innsbruck, Österreich

**Keywords:** Psoriasis, Retinoid, PUVA, Phosphodiesterase-4-Inhibitor, Lebensqualität, Psoriasis, Retinoids, PUVA therapy, Phosphodiesterase‑4 inhibitors, Quality of life

## Abstract

Es handelt sich um eine Fallserie von insgesamt 8 Patienten mit palmoplantarer Pustulose. Diese Patienten waren an unserer Psoriasisambulanz der dermatologischen Abteilung der Uniklinik Innsbruck mit dem Phosphodiesterase-4-Inhibitor Apremilast für zumindest 2 Monate behandelt worden. Das klinische Ansprechen wurde mit einem IGA(Investigator’s Global Assessment)-Score über die Dauer der Therapie bzw. mehrere Monate hin verglichen und dokumentiert. Die palmoplantare Pustulose zeichnet sich durch ihren starken negativen Einfluss auf die Lebensqualität der Betroffenen sowie durch ihre Therapieresistenz und Rezidivfreudigkeit aus. Therapieoptionen sind relativ rar bzw. nicht zugelassen. Apremilast ist eine gute und sichere Therapieoption bei palmoplantarer Pustulose.

## Abstract (en)

This is a case series of 8 patients with palmoplantar pustulosis. These patients were treated with the phosphodiesterase‑4 inhibitor apremilast at our psoriasis outpatient clinic at the dermatological department of the University Hospital Innsbruck and we compared and documented the clinical response using an Investigator’s Global Assessment (IGA) score over several months. This disease is characterized by its strong negative impact on the quality of life in affected patients, and by its resistance to therapy and its high relapse rate. Therapy options are relatively rare or off label. Apremilast is a safe and effective therapeutic approach in palmoplantar pustulosis.

Wir berichten retrospektiv über 8 Patient*innen (5 detailliert und 3 im Kurzüberblick) mit einer histologisch gesicherten Pustulosis palmoplantaris (PPP), die unter Therapie mit Apremilast stehen/standen, objektiviert durch den PPP-IGA (Investigator’s Global Assessment: 1–5, frei/fast frei/mild/moderat/schwer) zu Woche 0 und Woche 12 (zum Teil Woche 52). Alle 8 Patient*innen wurden zuvor mit UV-Therapie sowie systemischen Retinoiden bzw. auch in Kombination mit UV-Therapie behandelt. Bei 3 Patient*innen wurden eine Therapie mit Methotrexat, bei einer Patientin auch Infliximab – jeweils bis zum Wirkverlust – durchgeführt. Wir führen hier alle Patienten unserer Lichttherapieambulanz auf, die an einer histologisch bestätigten PPP leiden. Die Dauer bis zum Ansprechen unter Apremilast, Dauer bis zum Abbruch, Ursache des Abbruchs, Raucheranamnese, Komorbiditäten bzw. demografische Daten werden in Tab. 1 zusammengefasst.

**Table 1:** 

	Apremilast-Patienten mit Pustulosis palmoplantaris							
Pat	Alter	Alter bei ED	Sex	Raucher	Arthritis	Pso	Response	Apremilast-Abbruch (Therapiedauer/Grund)
1	71a	61a	W	Ja	Nein	Nein	Nach 2 Wochen	Nein (laufend seit 56 Monaten)
2	67a	59a	W	Nein	Nein	Nein	Nach 3 bis 4 Wochen	Nein (laufend seit 50 Monaten)
3	74a	34a	W	Ja	Nein	Nein	Nach 3 bis 4 Wochen	Nein (laufend seit 10 Monaten)
4	45a	43a	M	Ja	Nein	Nein	Nach 5 Wochen	Nein (laufend seit 8 Monaten)
5	45a	43a	M	Ja	Ja	Nein	Nach 3 Wochen	Nein (laufend seit 7 Monaten)
6	54a	53a	W	Ja	Nein	Nein	Nach 3 Wochen	Ja (nach 12 Monaten: Gewichtsverlust)
7	41a	30a	M	Nein	Ja	Nein	Nach 4 Wochen	Ja (nach 15 Monaten: Wirkungsverlust)
8	60a	56a	M	Ja	Ja	Ja	Nach 2 Wochen	Ja (nach 2 Monaten: anhaltende Diarrhö)

*Pat* Patient, *a* Jahre, *ED* Erstdiagnose, *Sex* Sexualität, *M* männlich, *W* weiblich, *Pso* Psoriasis

## Anamnese, Befund, Therapie und Verlauf

### Patientin 1 (71 Jahre, Raucherin, keine Psoriasis vulgaris)

Schwere PPP seit 10 Jahren, starke Schmerzen und Juckreiz, keine Gelenkbeteiligung.

#### Vortherapien.

Topische Therapie, PUVA-Therapie, Acitretin + PUVA, Methotrexat und Infliximab.

#### Beginn Apremilast.

Oktober 2015 (Observation laufend, zuletzt 06/2020).

Ansprechen von Schmerzen und Juckreiz innerhalb von 2 Wochen, langsame Rückbildung von Pusteln und Erosionen. Erytheme und Schuppung bestehen in wechselnder Ausprägung, nur intermittierend milder Juckreiz. Keine Nebenwirkungen. Die Patientin ist weiterhin sehr zufrieden mit nur 1 Schub in 5 Jahren, der durch intermittierenden Zusatz von UV-Therapie kontrolliert werden konnte.

PPP-IGA: Woche 0: 5, Woche 12: 3, Woche 52: 3.

### Patientin 2 (67 Jahre, Nichtraucherin, keine Psoriasis vulgaris)

Moderate PPP seit 8 Jahren mit Streuung, Juckreiz, keine Gelenkbeteiligung.

#### Vortherapien.

Topische Therapie, Bade-PUVA, Acitretin + PUVA, Methotrexat, Fumarsäure.

#### Beginn Apremilast.

November 2015 (Observation laufend, zuletzt 01/2020).

Rasches Ansprechen ohne Nebenwirkung. Die Patientin ist langfristig erscheinungsfrei (Therapiedauer bislang 5 Jahre). Ein selbstständiger Reduktionsversuch auf 1‑mal täglich führte zum Rezidiv.

**Figure 1:**
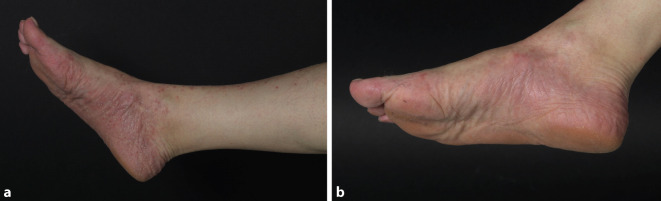

PPP-IGA: Woche 0: 3–4, Woche 12: 0, Woche 52: 0 (Abb. 1a, b).

### Patientin 3 (74 Jahre, Raucherin, keine Psoriasis vulgaris)

Moderate PPP-Erstmanifestation vor 40 Jahren, Rezidiv seit 3 Jahren, keine Gelenkbeteiligung.

#### Vortherapien.

Topische Therapie, Bade-PUVA, Acitretin + PUVA.

#### Beginn Apremilast.

Juli 2019 (Observation laufend, zuletzt 05/2020).

Initial Diarrhö für 3 Wochen, jedoch rasches, fast vollständiges Ansprechen.

PPP-IGA: Woche 0: 3–4, Woche 12: 0–1.

### Patient 4 (45 Jahre, Raucher, keine Psoriasis vulgaris)

Schwere PPP seit 2 Jahren, Schmerzen, keine Gelenkbeteiligung.

#### Vortherapien.

Topische Therapie, PUVA, Acitretin + PUVA, Methotrexat.

#### Beginn Apremilast.

Oktober 2019 (Observation laufend, zuletzt bis Juni 2020).

Kontinuierliches Ansprechen der Pusteln und Erosionen sowie der Schmerzen, aktuell Erytheme und Schuppen, Patient zufrieden und voll arbeitsfähig, keine Nebenwirkungen.

PPP-IGA: Woche 0: 5, Woche 12: 3.

### Patient 5 (45 Jahre, Raucher, keine Psoriasis vulgaris, Nagelpsoriasis, Arthritis)

Schwere PPP seit 18 Monaten, Juckreiz und Schmerzen, Verdacht auf Gelenkbeteiligung (Oligoarthritis, DD [Differenzialdiagnose]: Gicht), Nagelbeteiligung, Raucher, Alkoholabusus.

#### Vortherapien.

Topische Therapie, Bade-PUVA, Acitretin + PUVA.

#### Beginn Apremilast.

Dezember 2019 (Observation laufend, zuletzt bis Juni 2020).

**Figure 2:**
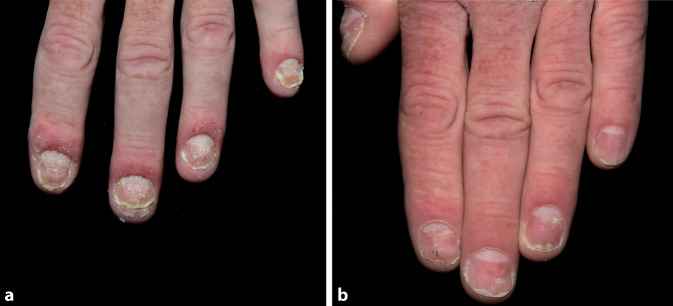

Rasches Ansprechen ohne Nebenwirkungen, gutes Ansprechen der Nagelbeteiligung (Abb. 2a, b) sowie auch der Gelenkschmerzen.

PPP-IGA: Woche 0: 4, Woche 12: 1.

Die retrospektiv analysierten Patient*innen zeigten alle ein rasches (innerhalb der ersten 2 bis 5 Wochen) Ansprechen. Allerdings mussten wir Apremilast bei 2 weiteren Patient*innen wegen Nebenwirkungen (Frau, 54 Jahre, nach über 1 Jahr trotz IGA-PPP von 0–1 wegen gastrointestinaler Nebenwirkungen und Gewichtsverlust; Mann, 60 Jahre, vorbestehend assoziierte Psoriasis vulgaris, nach 2 Monaten wegen anhaltender Diarrhö und Gewichtsverlust) und bei 1 Patienten wegen sekundären Wirkverlustes (Mann, 41 Jahre, nach 15 Monaten) absetzen. Somit muss auch auf die relativ hohe Abbruchrate der Therapie mit Apremilast von 37,5 % (3 von insgesamt 8 Patient*innen) hingewiesen werden.

## Diskussion

Die Pustulosis palmoplantaris (PPP) ist eine chronische pustulierende Dermatose, die sich im Verhältnis zur betroffenen BSA („body surface area“) überproportional negativ auf die Lebensqualität auswirkt. Ob es sich um eine eigenständige Entität oder einen Subtyp der Psoriasis vulgaris handelt, bleibt umstritten. Interessanterweise leiden nur zwischen 16 und 30 % der Patient*innen gleichzeitig an Psoriasis vulgaris, dies ist im Vergleich zu anderen Formen der pustulierenden Psoriasisformen wie der generalisierten Psoriasis pustulosa (Assoziation mit Psoriasis vulgaris bei 54 %) oder der Acrodermatitis continua Hallopeau (46 %) geringer [1]. Die Assoziation mit Psoriasisarthritis liegt ebenfalls bei etwa 30 %. Es wird vermutet, dass Infektionen und Lebensstilfaktoren, insbesondere Nikotinabusus (80 % der Patient*innen sind Raucher), einen Einfluss auf die Verschlechterung dieser Krankheit haben [2]. Pustulosis palmoplantaris ist eine seltene Erkrankung mit einer Prävalenz von 0,01–0,05 %. Sie tritt deutlich häufiger bei Frauen auf (77 %), das durchschnittliche Alter zu Beginn der Erkrankung liegt bei 45,3 Jahren [3].

Die Behandlung der Patient*innen mit PPP ist schwierig, die Ansprechraten sind im Vergleich zur Psoriasis vulgaris deutlich niedriger. Die gängigen Therapien beinhalten indifferente und differente Topika, die auch bei der Psoriasis vulgaris verwendet werden, UV-Therapie sowie systemische Retinoide, oft in Kombination mit UV-Therapie, oder Methotrexat. Insgesamt ist die Datenlage zur Behandlung der PPP schwach, wobei einzelne Kasuistiken existieren, die ebenfalls ein gutes Ansprechen der PPP unter Apremilast beschreiben [4–6].

Apremilast ist ein Phosphodiesterase-4-Inhibitor, der intrazellulär die Produktion von Entzündungsmediatoren wie Interleukin(IL)-17, Tumornekrosefaktor‑α, IL-23 und entzündungshemmenden Mediatoren reguliert, die auch an der Pathogenese von Psoriasis beteiligt sind [7]. Es hemmt PDE(Phosphodiesterase)4-Isoformen aus allen 4 Unterfamilien (A1A, B1, B2, C1 und D2), in Monozyten und T‑Zellen wird das intrazelluläre cAMP (zyklisches Adenosinmonophosphat) erhöht und der Transkriptionsfaktor 1 aktiviert, während die NF-κB(nuclear factor „kappa-light-chain-enhancer“ of activated B‑cells)-Transkriptionsaktivität inhibiert wird. Apremilast reduziert die Interferon-α-Produktion und inhibiert die T‑Zell-Zytokinproduktion, hat jedoch wenig Einfluss auf die B‑Zell-Immunglobulinsekretion [8].

Die Häufigkeit schwerwiegender kardialer Ereignisse, maligner Erkrankungen und Depressionen nahm bei langfristiger Apremilast-Exposition nicht zu. Die Rate schwerer Infektionen blieb auch bei langfristiger Therapie niedrig. Initiale gastrointestinale Symptomatik ist die häufigste Nebenwirkung. Unter der Therapie mit Apremilast ist keine routinemäßige Laborüberwachung laut Produktbeschreibung erforderlich.

Insgesamt sehen wir, wie auch schon in einzelnen Kasuistiken [4–6], aber auch in einer multizentrischen retrospektiven Analyse mit 347 Patient*innen [9] beschrieben, ein großes Potenzial dieses therapeutischen Ansatzes bei palmoplantarer Pustulose. Das ausgeglichene Geschlechterverhältnis ist wahrscheinlich der geringen Fallzahl geschuldet. Der retrospektive Charakter und die geringe Fallzahl von 8 Patient*innen sind die Schwachstellen dieser Analyse.

Prospektive Studien wären zur weiteren Klärung wünschenswert.

## Fazit für die Praxis

- Bei Therapieversagen auf Topika bzw. lokalisierte PUVA in Kombination mit Retinoiden ist der Phosphodiesterase-4-Inhibitor Apremilast eine gute und sichere (wenn auch nicht zugelassene) Therapieoption.
- Aufgrund der niedrigen Rate an Infektionen, kardialer Ereignisse und maligner Erkrankungen ist Apremilast auch für Patienten mit spezifischen Vorerkrankungen eine mögliche Alternative.
- Anhaltende Diarrhö über mehrere Wochen mit Gewichtsverlust (trotz supportiver Maßnahmen) sowie Wirkverlust zählen zu den häufigsten Gründen für den Therapieabbruch.
